# The experiences and perceptions of personhood for people living with dementia: A qualitative evidence synthesis protocol

**DOI:** 10.12688/hrbopenres.12845.1

**Published:** 2018-06-05

**Authors:** Niamh Hennelly, Adeline Cooney, Catherine Houghton, Eamon O'Shea

**Affiliations:** 1 Centre for Economic and Social Research on Dementia, National University of Ireland, Galway, Ireland; 2 Centre for Teaching & Learning, Maynooth University , Maynooth, Ireland; 3 School of Nursing and Midwifery, National University of Ireland, Galway, Galway, Ireland

**Keywords:** Dementia, personhood, self-identity, systematic review, qualitative synthesis

## Abstract

**Background: **Personhood in dementia is concerned with treating people living with dementia with dignity and respect, in a manner that supports their sense of self. It focuses on treating the person living with dementia as a person first and foremost. Supporting personhood in dementia is the key goal of person-centred care. Existing qualitative research examines what personhood means to the person living with dementia and explores what is important to their personhood and sense of self. However, to date little work has focused on synthesising these studies.

**Methods**: This is a protocol for a qualitative evidence synthesis of personhood in dementia. The review examines qualitative peer-reviewed research of the perspectives and experiences of personhood for people living with dementia. A systematic search will be carried out on eight electronic databases and supplemented by other purposeful literature search methods. Title and abstract screening, and full text screening will be carried out by two authors independently. Included studies will be critically appraised. Thematic synthesis will be conducted on all of the included studies. Confidence in the review findings will be assessed using GRADE CERQual.

**Discussion:** The findings from this synthesis will be useful to health care providers and policy makers seeking to understand what personhood means for people living with dementia. The findings will also inform optimal service provision, as well as outcome measures in dementia.

**PROSPERO registration**: CRD42017076114 (21/11/2017)

## Introduction

In 2015, the number of people living with dementia was estimated at 46.8 million globally. This is predicted to increase significantly to 131.5 million by 2050 ( Prince *et al*., 2015). Dementia impacts profoundly on the person living with dementia but also has significant social effects, not least on family and friends. Until very recently, the biomedical model has dominated our thinking on dementia ( Cahill, 2018). It viewed people living with dementia as lacking rationality and capacity and by consequence having lost personhood ( Baldwin *et al*., 2007; Caddell & Clare, 2010; Small *et al*., 1998; Surr, 2006). Kitwood (1997) posits that treating the person living with dementia as if they are no longer a person, as if they had lost personhood, exacerbates the impact and the progression of the dementia.

Research from social psychology into the self in dementia concurs with Kitwood’s theory and shows the critical role that people around the person living with dementia play in supporting their sense of self and social roles ( Sabat & Harré, 1992; Sabat & Collins, 1999). The implications of these findings on dementia care have resulted in a gradual paradigm shift from a biomedical model to a biopsychosocial model of dementia care ( O'Shea & Carney, 2016). This model takes a whole-person approach to understanding dementia and examines a wide range of factors likely to impact on the person’s subjective experience, including influences from biology, society, economics, psychology and the environment.

Person-centred care is firmly rooted in the biopsychosocial model and is centred on recognising and supporting personhood in dementia ( Brooker, 2007; Edvardsson *et al*., 2008; Hughes & Beatty, 2013). McCormack *et al*. (2012) argue that evidence and research into personhood is critical to the application of best practice in person-centred care. While there are existing studies on personhood in dementia and in the related areas of sense of self and self-identity, very little of that research has been systematically reviewed and synthesised. Moreover the voice of the person living with dementia in relation to personhood has not been heard as strongly as one might expect. This review aims to systematically search the literature to examine what personhood means to people living with dementia and, in particular, what they define as its most important constituent parts.

## Personhood in dementia

Primarily, personhood in dementia is concerned with how the person living with dementia perceives themselves ( Sakamoto *et al*., 2017) and how this can be supported by those around them in relation to their own being and identity. For example, Leibing (2008) explains how personhood is concerned with “that which really matters” to us as people and how important it is for people living with dementia that care practices does not “diminish someone’s ‘humanness” (2008, p.183). Selfhood and self-identity are intrinsically linked with, and viewed as, core elements of personhood. McCormack *et al*. define personhood as “a sense of self-identity maintained by relationships” (2012, p.286). Personhood in dementia is predominantly conceptualised as relational and socially constructed placing it within the domain of roles, relationships and social interaction ( Tolhurst *et al*., 2014). Hennelly & O’Shea (2017), found synonyms for personhood to encompass an even wider range of terms such as connection, dignity, holistic, identity, person-centred, relationships, role, respect and self.

There are a number of existing reviews and syntheses on the experiences of people living with dementia ( Ablitt *et al*., 2009; Eriksen *et al*., 2016; Górska *et al*., 2018; La Fontaine & Oyebode, 2014; Steeman *et al*., 2006; von Kutzleben *et al*., 2012; Wadham *et al*., 2016; Wolverson *et al*., 2016). However, there are few reviews of personhood in dementia, particularly ones that focus exclusively on the direct experiences of the person living with dementia. Caddell & Clare (2010) conducted a comprehensive systematic review of both qualitative and quantitative research to investigate self and identity in dementia, but they did not include any type of synthesis. They found that the majority of studies supported the persistence of self in people living with dementia, including people with severe dementia. Our review will build on their work by synthesising existing qualitative research on personhood from the perspective of the person living with dementia, exploring their experiences and perceptions of personhood, self-identity, sense of self and selfhood. However, the review will not seek to examine the evidence in relation to the existence of self, as Caddell & Clare (2010) have already examined this issue.

## Rationale

In his theorising about personhood in people living with dementia, Kitwood (1997) identified five psychological needs experienced by people living with dementia, namely the need for comfort, attachment, identity, occupation and inclusion. He also talked about the relationship aspect of dementia and the importance of inter-personal relations in building and maintaining personhood. Maintaining personhood in the face of declining cognitive powers presents a huge challenge to care systems in all countries. Brooker’s (2004) response was to set out a person-centred care approach comprising of four key elements: a value base; an individualised approach emphasising uniqueness; adopting the perspective of the individual; and providing a supportive social environment.

Reviews of person-centred care in dementia have been undertaken ( Chenoweth *et al*., 2015; Houghton *et al*., 2016; Kim & Park, 2017). However, there are few reviews of research on personhood. In carrying out this review we will assess how much research has been conducted on personhood, what the quality of this work is, what the results of those studies tell us and, if feasible, we will synthesise these studies to provide a more comprehensive view of personhood in dementia. The findings from the synthesis will be useful in designing, implementing and assessing future work on personhood, in relation to person-centred care for people living with dementia in all care settings. Findings will also be useful in informing future practice, regulation and policy in dementia care, including the measurement of personhood-related outcomes.

## Protocol

### Aims and objectives

This review aims to examine the experiences and perceptions of personhood for people living with dementia in various dementia care settings using qualitative evidence synthesis. The objectives of the review are to:

1. Describe the experiences and perceptions of personhood for people living with dementia living at home and across home, community and long-stay care settings.
2. Examine the potential implications of this synthesis for practice, regulation and policy in dementia care.

## Methods

### Eligibility criteria

***Inclusion criteria.***

**Types of studies:**

Primary research studies which are qualitative and mixed-methods will be considered for inclusion. The design methods (e.g. semi-structured interviews or observation) and the analysis (e.g. thematic analysis or grounded theory) must be clearly reported and must be qualitative to be included in the review. Mixed-methods studies will be included if the qualitative element is clearly reported. All studies must be peer-reviewed articles.

**Types of participants:**

Study participants are people with any type of dementia, including Alzheimer’s disease, Lewy Body dementia, vascular dementia, Pick’s Disease, Huntington Disease, Frontotemporal Dementia, or Creutzfeldt-Jakob disease. Studies focusing on people with early to late-onset dementia of all ages will be included. A formal diagnosis of dementia will not be necessary. Studies will be included once the study author(s) have stated that the participants have dementia. If the study also contains participants who do not have dementia, then it must be possible to extract the views and expressions of the participants with dementia within such studies. Otherwise, these studies will be excluded.

**Phenomenon of interest:**

The phenomenon of interest in this study are the experiences and perceptions of personhood for people living with dementia which includes studies of the self, self-identity, selfhood and sense of self. If studies examine personhood along with another concept in dementia then it must be possible to extract the information specific to personhood in order for the study to be included in the synthesis.

**Comparison:**

Perspectives of personhood in dementia will be solely from people living with dementia. There is the potential for subgroup analysis within this population, for example, analysis of personhood in relation to care provided in different care settings.

**Evaluation:**

The results of this synthesis will be useful in guiding practice and policy which aim to support personhood in dementia. The results will be useful for both implementation of such practices and examining outcomes in dementia.

**Settings:**

Studies will be in three types of settings: home care, day care and long-stay care. This review will include studies from any country and in any language.

***Exclusion Criteria.***

**Types of studies:**

- Quantitative research
- Mixed-methods studies that do not report their qualitative methods nor present qualitative findings
- Literature reviews and editorials
- Non-peer reviewed items including: grey literature, reports and theses
- Studies which collect data qualitatively and analyse it quantitatively (e.g. descriptive analysis) will also be excluded.
- Studies where the full text is not available

**Types of participants:**

- Studies where the person living with dementia is not a participant
- Studies where the participants have a mild cognitive impairment
- Studies where it is not possible to extract the views of the person living with dementia

**Phenomenon of interest:**

- Research on artistic expressions of personhood including interpretation of texts, art or film
- Studies which examine interventions to support personhood including for example, reminiscence, person-centred care interventions, self-management interventions, art therapies etc.
- Studies which examine other elements of subjective experiences in dementia such as coping, social relationships, dignity and meaning-making along with personhood where it is not possible to extract the data on personhood

### Search methods for identification of studies

This review will use a combination of systematic searching of the literature using electronic databases and other search methods including purposive sampling of papers using key citations and hand searching of references ( Booth, 2016).

It will search the electronic databases listed in Table 1.

**Table 1.** Electronic databases.

1.	Scopus
2.	Web of Science
3.	EBSCO – CINAHL and AgeLine
4.	Sociological Abstracts
5.	PsycINFO
6.	Medline
7.	Embase
8.	Cochrane

These databases have been selected, in consultation with an expert librarian, in order to source peer-reviewed articles across a number of disciplines including nursing, psychology, social care and social gerontology. The search will be conducted by one author (NH) in all databases over one day. In addition, this review will use the Google Scholar’s *Cited by* option to include all papers referencing Kitwood & Bredin (1992) and Sabat & Harré (1992) which are two seminal works on personhood in dementia. Reference searches of the included papers will also be conducted. This involves both manual backward searching of their references and forward searches using Google Scholar’s *Cited by* option ( Booth, 2016). The search will not be limited to particular geographic locations and will include papers in any language. The search will cover from 1985 to present, starting several years prior to the publication of Kitwood & Bredin (1992) and Sabat & Harré (1992).

***Electronic Search.*** A summary of the electronic search strings is presented in Table 2.

**Table 2.** Search strings.

	personhood OR person*hood OR selfhood OR self*hood OR self-identity OR self*identity OR identity OR “sense of self” OR self (Title and abstract)
AND	dementia OR Alzheimer* OR “Lewy Bod*” OR “vascular dementia” OR pick* OR Huntington* OR frontotemporal OR Creutzfeldt-Jakob OR “cognitive impairment”(Title and abstract)
AND	qualitative OR “mixed*method*” OR narrative OR phenomenol* OR ethnograph OR ethnonursing OR ethnological OR questionnaire OR “grounded*theory” OR “case*stud*” OR “action*research” OR “focus*group*” OR thematic OR construction* OR hermeneutic OR heurist* (Title and abstract)

Certain terms will be truncated, for example dement* or Lewy Bod*, to ensure all spellings are captured. MeSH terms will be used for Medline. The use of title and abstract will depend on the individual databases. This review will report results of searching, screening and included studies using the PRISMA flowchart ( Moher *et al*., 2009).

### Screening

All references will be imported into Endnote and duplicates removed. Two authors (NH and AC) will screen titles and abstracts independently, using Covidence. When there is no abstract or it is not possible to determine whether to include an article or not, the full text of the article will be retrieved. One author (NH) will review all full-text articles, two other authors (CH and EOS) will share second screening of all full-text articles. Disagreement between authors will be discussed and if required, in consultation with all authors. If necessary, we will contact authors of potential included studies for further clarification and information. If the review retrieves more than 40 eligible papers then the CART framework will be used to determine what articles to include ( Aslam *et al*., 2017; Tennison, 2013). This encompasses examining the completeness, accuracy, relevance and timeliness of the studies ( Aslam *et al*., 2017; Tennison, 2013).

### Data extraction and management

Full text articles will be imported and managed within NVivo. Data extraction and the thematic synthesis will be facilitated within NVivo ( Houghton *et al*., 2017). The data extraction form will be created within NVivo using categories such as: author, year, location, methodology, ethics, data collection, results/findings including participant quotes etc.

***Assessment of methodological limitations in primary studies.*** The included studies will be assessed for quality using the Critical Appraisal Skills Programme tool. This includes examining: the aims of the study, methodology, research design, recruitment strategy, data collection, relationship between researcher and participant, ethics, data analysis, findings and the value of the research. How the study is reported may not be reflective of how the study was conducted and therefore this assessment will not be used to exclude any studies from the synthesis ( Dixon-Woods *et al*., 2007). The appraisal will be carried out by one author (NH) and reviewed by a second (EOS).

### Data synthesis

The field of qualitative evidence synthesis is wide and varied ( Barnett-Page & Thomas, 2009). Qualitative synthesis differs to narrative and systematic literature reviews as it aims to go beyond solely describing qualitative studies to developing new interpretations or explanations of these studies’ findings ( Barnett-Page & Thomas, 2009). This review will synthesise the included qualitative studies using thematic synthesis ( Thomas & Harden, 2008). This is a three step process which starts with line by line coding of primary data from the included studies in order to develop descriptive themes and generate broader analytical themes ( Thomas & Harden, 2008). This process will be conducted within NVivo with guidance from a previous synthesis on how best to use the coding software ( Houghton *et al*., 2017). Thematic synthesis was chosen because the final result is particularly useful for providing information for policy and practice ( Barnett-Page & Thomas, 2009). One author (NH) will carry out the thematic synthesis with continuous input from the other three authors at each stage. Using NVivo to carry out the whole process from line by line coding to analytical themes provides for transparency and clarity in the synthesis process. All of the authors will read and make contributions to the final paper. As this is a qualitative evidence synthesis, no statistical analysis is planned on the papers selected for the review.

***Sub group analysis and heterogeneity.*** Given the significant impact of setting on the dementia experience, a sub group analysis between home and residential care settings will be carried out. Other sub group analysis many include experience of personhood in formal care provision, people with young onset dementia, gender or ethnicity. Such groups will be determined inductively through the synthesis.

### Assessment of confidence in the review findings

***Appraisal of review findings.*** This review will use the GRADE CERQual approach, to appraise the review findings, which involves examining four main elements: the limitations of included studies, how relevant the studies are to the review question, the coherence of the review finding and how adequate that data is in supporting the review finding. This will include a sensitivity analysis to examine the contribution of the poorer quality studies to the overall findings ( Houghton *et al*., 2017; Thomas & Harden, 2008). The appraisal of review findings will be carried out by one author (CH) and reviewed by a second (NH).

***Reporting.*** This review will be reported in line with the ENTREQ guidelines ( Tong *et al*., 2012). A completed PRISMA-P checklist is available as Supplementary File 1.

***Dissemination of findings.*** Findings will be submitted to a peer-reviewed journal for publication. The findings will also be integrated into a wider study being conducted at NUI Galway, also funded by the Health Research Board of Ireland (HRB), on resource allocation processes in dementia care provision. The findings will also be shared with stakeholders, including people with dementia, as part of the commitment to Public Patient Involvement (PPI) at the Centre for Economic and Social Research on Dementia in NUI Galway.

***Study status.*** This study is currently underway. Title and abstract screening has taken place. Full-text screening is ongoing. Data extraction has not yet taken place.

## Discussion

Personhood, and its realisation in person-centred care, is nowadays part of the narrative, if not always the reality, of care for people living with dementia. While it appears that we have come a long way from Kitwood’s (1993) early theorising about personhood to a clearer understanding of the practical application of the model, personhood remains a contested space within the dementia literature. Moreover, an absence of clarity with respect to the conceptualisation and actualisation of personhood within policy documents, for example the Irish National Dementia Strategy ( Hennelly & O'Shea, 2017), makes it difficult to assess how person-centred care can be enhanced for people living with dementia through changes in current practice. The challenge is to provide the information, knowledge, incentives and resources for personhood to take hold in dementia care across all care settings. The purpose of this protocol is to systematically assess peer-reviewed qualitative studies of personhood in dementia from the perspective of people living with dementia themselves. This will help give direct voice and influence to people living with dementia in shaping the narrative with respect to personhood and person-centred care in dementia. The outcomes of the planned synthesis will be useful as an aid to developing an experience-led appreciation and understanding of the key elements of personhood in dementia. The results will be important in planning care and supports for people living with dementia that focus on, and support, personhood-focused care, as interpreted by people living with the disease. Structuring person-centred care around what matters to people living with dementia increases the potential of enhancing their care and quality of life. Additionally, these results will be useful for evaluation, regulation and outcome assessment in dementia care. They will also act as a benchmark for people living with dementia and their family carers in relation to personhood ideals and attributes.

## Protocol registration

Registered with International Prospective Register of Systematic Reviews (PROSPERO) no. CRD42017076114 on the 21 ^st^ November 2017. The following amendments were made to the original protocol: the electronic search string was edited and the expected completion date of the review was extended. Both versions of the protocol are available on www.crd.york.ac.uk/prospero. Any further amendments to the protocol will be updated on PROSPERO.

## Data availability

No data is associated with this article

## Supplementary material

Supplementary File 1 – Completed PRISMA-P checklist
