# Supplementary material for: The experiences and perceptions of personhood for people living with dementia: A qualitative evidence synthesis protocol
Source: HRB Open Res. 2018 Jun 5;1:18. [Version 1] doi: 10.12688/hrbopenres.12845.1 (PMC7219284; doi:10.12688/hrbopenres.12845.1)
Supplement: Peer review history

# Peer review: Reviewer response for version 1

**DOI:** 10.21956/hrbopenres.13907.r26259

**Published:** 2018-06-28

**Authors:** Deborah O'Connor

**Affiliations:** 1 School of Social Work, University of British Columbia (UBC), Vancouver, BC, Canada

Thank you for inviting me to review this proposed study. As the authors note, there is a growing body of research now linked to how people with dementia experience dementia, much of which is grounded within the personhood literature. However, to the best of my knowledge, this literature has not been synthesized. Certainly, as a researcher, I would welcome this and look forward to seeing the end results of this study.

 Generally, I see this proposed study as well-developed and straight-forward. I do have a few relatively minor questions/comments for the authors to consider:

**Criteria for inclusion/exclusion:**  

1. The authors note that: “Studies focusing on people with early to late-onset dementia of all ages will be included. A formal diagnosis of dementia will not be necessary”. I have two questions here. First,  how will the authors ascertain that this research has indeed focused on persons with dementia? Second, while the authors note here they will include early to late-onset, later in the protocol they indicated that studies where participants have a mild cognitive impairment will be excluded. Can these two, potentially conflicting statements, be clarified? Perhaps the authors are referencing MCI as a diagnosis? If so, how does this align with not requiring a diagnosis of dementia?
2. In terms of selection, the authors indicate that they will examine “all papers referencing Kitwood & Bredin (1992) and Sabat & Harré (1992) which are two seminal works on person- hood in dementia”. I would suggest expanding this to include Kitwood 1997 as often this is cited in lieu of the 1992 paper. As well,  anything referencing Sabat is probably relevant as well.
3. The authors indicate they will only address peer-reviewed publications. While on the one hand I understand this, it would be useful to explicate why other sources are being excluded and to recognize the limitations associated with this decision to exclude. This is particularly pertinent to the decision to exclude autobiographical accounts (which arguably, a case could be made for them to be considered case studies). Similarly, there are a number of policy papers that have attempted to bring forward the perspectives of those with dementia, can the authors explain why these will be excluded including acknowledging the limitations of their exclusion.

**Analysis and Synthesis**

 When asked if sufficient details about methodology were provided that this study would be replicable, I responded ‘partly’. The rationale behind my hesitant response is related to three issues that I think could usefully be further developed to increase replicability:

1. Initially I wondered if only studies in English would be considered, but later noted that non-English papers will also be included. How do the authors intend to handle non-English papers?
2. In terms of developing a protocol that is replicable, it would be useful if the author’s identified the questions or topics that they will use to insure a systematic reading of each paper. I recognize that some broad questions have been identified (i.e. Study quality) and I suspect there are some implicit questions, but as a reader it would be helpful if these were more explicitly operationalized into some sort of a reading protocol. This would facilitate contrast and comparison?
3. Picking up on my previous comment: I think there are some aspects related to a synthesis of this body of research that would be of particular value to address. One of these is culture: for example, how does culture inform each study including sense-making of perceptions/experiences of personhood? I note that the authors’ state that “Other sub group analysis many[*] include experience of personhood in formal care provision, people with young onset dementia, gender or ethnicity”. Personhood has been critiqued for it’s cultural relevance. From my perspective, some incorporation of culture into this synthesis would increase its value. Hence, I would recommend that the tentative ‘ *may*’ be changed to a more intentional ‘ *will* ‘ be included as a sub-group analysis. I would also encourage broadening beyond ethnicity to consider for example, race and sexual orientation. 

I hope these comments are helpful and as previously noted, I look forward to reading this synthesis!

  * Note need to edit this sentence as this is a direct quote: I’m assuming the ‘many’ is supposed to read ‘may’.

I confirm that I have read this submission and believe that I have an appropriate level of expertise to confirm that it is of an acceptable scientific standard.

# Peer review: Reviewer response for version 1

**DOI:** 10.21956/hrbopenres.13907.r26262

**Published:** 2018-06-21

**Authors:** Steven R. Sabat

**Affiliations:** 1 Georgetown University, Washington, DC, USA

I indicated that the study is "partly" appropriate for the research question because it is appears to be limited to peer reviewed work. Even though there is a statement to the contrary within the protocol, there seems to be an omission of important references. For example, the voices of people with dementia, such as those of Kate Swafford, Richard Taylor, and Christine Bryden, for example, who have written books on their experience of dementia are omitted in this study, but should not be. The fact that their work is not peer reviewed should not disqualify their voices from the study because, after all, their voices are precisely those that the authors seek to hear. Likewise, books such as Snyder's *Speaking Our Minds: What it's Like to have Alzheimer's *should be included as well for the same reasons as mentioned above. All modesty notwithstanding, my own work in book form is germane as well. The perspective of people with dementia can be found in great detail in sources other than peer reviewed journals and although the authors have noted that non-peer reviewed items are included, the above authors' works are not included in the reference section and, I believe, inclusion of those works would add to the authors' protocol especially because they are so clearly and correctly concerned with the perspective of people living with dementia (see Discussion section).

 Having said that, it is excellent that the studies reviewed will include the settings of home care, day care, and long-term care. This is especially important and a potential source of valuable information as are studies using a variety of methods (quantitative, qualitative, mixed, etc.). Likewise, I think that the "Phenomenon of Interest" is excellent for its inclusivity of a variety of expressions of personhood, such as art and social relationships, dignity, and meaning making for example. The authors are to be congratulated for the breadth of their protocol in this respect as well as exploring contributions from a number of different disciplines and databases and geographic locations. This, too, is evidence of the authors' understanding that knowledge can be gained from using a wide-angle lens, as it were, so as to include a variety of methods and venues of reporting information. The inclusion of a variety of data bases is also admirable.

 I appreciate the authors' intention to assess methodological limitations in primary studies. The assets and liabilities of methods of investigation is an important contribution to the study. The same is true of subgroup analysis involving people living in a variety of settings and people with varied gender and ethnicity. This is especially important because the "one size fits all" variety of understanding is terribly flawed in light of the idea of person-centered care.

 I agree wholeheartedly with the authors' goal of improving person-centered care by including the perspective of those very people. In the words of Christine Bryden, there should be "nothing about us without us" and this, as the authors clearly note, needs to be applied in all care settings so that it can be shown that doing so is not only beneficial for those diagnosed and their care partners, but also economically and socially beneficial for the country itself. The protocol is especially important and timely and I am very impressed with its scope and, of course, with the rather important rationale for improving further person-centered care for people living with dementia.

I confirm that I have read this submission and believe that I have an appropriate level of expertise to confirm that it is of an acceptable scientific standard.
